# Expression Status of PIWIL1 as a Prognostic Marker of Colorectal Cancer

**DOI:** 10.1155/2017/1204937

**Published:** 2017-05-29

**Authors:** Rui Sun, Chun-li Gao, Dong-hai Li, Bo-jun Li, Yan-hong Ding

**Affiliations:** ^1^Department of Oncology, The First People's Hospital of Lanzhou City, Lanzhou, Gansu 730050, China; ^2^Department of Gastroenterology, The First People's Hospital of Lanzhou City, Lanzhou, Gansu 730050, China; ^3^Department of Pathology, The First People's Hospital of Lanzhou City, Lanzhou, Gansu 730050, China

## Abstract

The PIWI-like protein 1 (PIWIL1) plays a crucial role in stem cell proliferation, embryogenesis, growth, and development, as well as differentiation and maturation in multiple organisms. The relationships between PIWIL1 expression and clinicopathological features of colorectal cancer (CRC) patients were analyzed by us. Survival analysis was performed using the Kaplan-Meier method and Cox's proportional hazards model. The high expression rate of PIWIL1 in the cancer tissue was obviously higher than that in the corresponding adjacent tissue. The expression of PIWIL1 was closely related to the tumor differentiation degree, infiltration depth, lymphovascular invasion, lymph node metastasis, and TNM stage. The Kaplan-Meier survival model suggested that the survival time of CRC patients in the high PIWIL1 expression group was notably lower than that in the low PIWIL1 expression group. High PIWIL1 expression suggests a poor prognosis for CRC patients, and PIWIL1 can serve as an important molecular marker for predicting the prognosis of CRC patients.

## 1. Introduction

Colorectal cancer (CRC) is one of the common malignancies of the gastrointestinal system. In 2012, the global morbidity and mortality of CRC were about 1.3606 million and 0.6939 million, respectively [1, 2]. With the emergence of the aging society and changes in the living pattern in China in the recent years, the morbidity and mortality of CRC in China have shown gradual increasing trends and now rank the fifth and the third, respectively [3]. Currently, the treatment of CRC is dominated by surgery, but the 5-year survival after surgery is only 50%. Therefore, investigating the molecular mechanisms of the genesis, development, invasion, and metastasis of CRC and searching for effective therapeutic targets of CRC is of great significance for improving the survival of patients.

Scholars believe that tumors derive from a small fraction of stem cells, based on the source of the tumor stem cell theory [4]. Stem cells possess highly proliferative, continuous self-renewing, and multipotential differentiation capacities [5]. These capacities of stem cells may be closely associated with the genesis, development, metastasis, and recurrence of tumors. The PIWI-like protein 1 (PIWIL1) is an important member of the Argonaute protein family, and it plays a crucial role in stem cell proliferation, embryogenesis, growth, and development, as well as differentiation and maturation in multiple organisms [6, 7]. In this study, we detected the expression of PIWIL1 in CRC and analyzed its relationship with the clinicopathological characteristics of patients with CRC.

## 2. Methods

### 2.1. Patients and Samples

During January 2007 and December 2010, 110 cases of CRC that underwent surgical resection at The First People's Hospital of Lanzhou City, along with the corresponding adjacent tissues, were collected. All of the patients had a clear pathological diagnosis as well as complete clinical data. There were 52 cases of colon cancer and 58 of rectal cancer. The age of the patients ranged from 34 to 75 years, with an average of 52.8 ± 10.5 years. Clinical staging was conducted in accordance with the tumor node metastasis (TNM) staging system formulated jointly by the American Joint Committee on Cancer (AJCC) and the Union for International Cancer Control (UICC) [8]. Twenty-three cases were stage I, 42 were stage II, and 34 were stage III. All patients with colorectal cancer did not receive chemotherapy, radiotherapy, or biotherapy preoperatively. All patients or their families signed the informed consent form, and this study was approved by the Ethics Committee of The First People's Hospital of Lanzhou City.

### 2.2. Immunohistochemistry

Serial sections of the paraffin specimens were made, followed by conventional dewaxing and hydration with xylene and ethanol; they were placed in the citrate antigen repair liquid for antigen retrieval and then rinsed twice with phosphate-buffered saline (PBS). Next, they were incubated in 1.5% H_2_O_2_-deionized water for 30 min and rinsed with distilled water. They were incubated in normal serum at 37°C for 15 min and then we added the primary antibody (rabbit anti-human PIWIL1 antibody, 1 : 500, ab135003; Abcam, Cambridge, UK; the primary antibody was replaced with PBS in the blank control group) and incubated in the refrigerator at 4°C overnight. After rinsing with PBS, the secondary antibody (1: 200; Shanghai Kaibo Biotechnology, Shanghai, China) was added, and the sections were incubated at 37°C for 60 min. We then allowed the color to develop with DAB for 30 min, by adding the peroxidase-labeled streptavidin dropwise, incubation at 37°C for 20 min, followed by mounting after counterstaining with hematoxylin.

We randomly selected 5 medium views (×200), and 200 tumor cells were counted in each view, with altogether 1000 cells being counted. The staining intensity was divided into the following: 0 point: no staining; 1 point: faint yellow staining; 2 points: claybank staining; and 3 points: dark brown staining; and the positive cell proportion was divided from 0 to 100%. In this study, the products of the staining intensity and the positive proportion being >0 was defined as PIWIL1 positive; otherwise, it was defined as negative. To sum up, PIWIL1 staining intensity of 0 and 1 point was deemed as low expression, while that of 2 and 3 points was regarded as high expression.

### 2.3. Reverse Transcription-Polymerase Chain Reaction (RT-PCR)

TRIzol reagent was utilized to extract the total RNA. The mRNA was reverse transcribed into cDNA according to the RT-PCR instructions, and the cDNA was used as the template for PCR amplification. The PCR reaction conditions were 94°C for 5 min, 94°C for 30 s, 56°C for 30 s, and 72°C for 40 s for 30 cycles, followed by 72°C extension for 5 min. The primer sequences of PIWIL1 were forward: 5′-TCACCTGAGCAAAGACAAC-3′ and reverse: 5′-TCCCGTAAAGGACAGTAAG-3′. The amplification products were separated on a 2% AGE and gel imaging, observation, and photo taking were performed under an ultraviolet lamp. Quantity One software (Bio-Rad Laboratories, Philadelphia, PA, USA) was utilized to analyze the gray value of the electrophoretic band, and GAPDH was used as the internal reference. The primer sequences of GAPDH were forward: 5′-GGAAGGTGAAGGTCGGAGTCA-3′ and reverse: 5′-GTCATTGATGGCAACAATATCCACT-3′.

### 2.4. Statistical Analyses

The relationship between the expression of PIWIL1 and the clinicopathological characteristics of CRC patients was analyzed through the chi-squire test. Rank sum test analysis was utilized to compare the mRNA levels between the two groups. The major outcome indexes were disease-free survival (DFS) and overall survival (OS), and the Kaplan-Meier method was utilized to calculate the survival curves of the CRC patients. The Cox proportional hazard regression model was adopted for multi-factor survival analysis. The SPSS 17.0 software package (SPSS Inc., Chicago, USA) was used to conduct statistical analysis, and a difference of *P* < 0.05 was deemed as statistically significant.

## 3. Result

### 3.1. The PIWIL1 Expression in CRC

**Figure 1:**
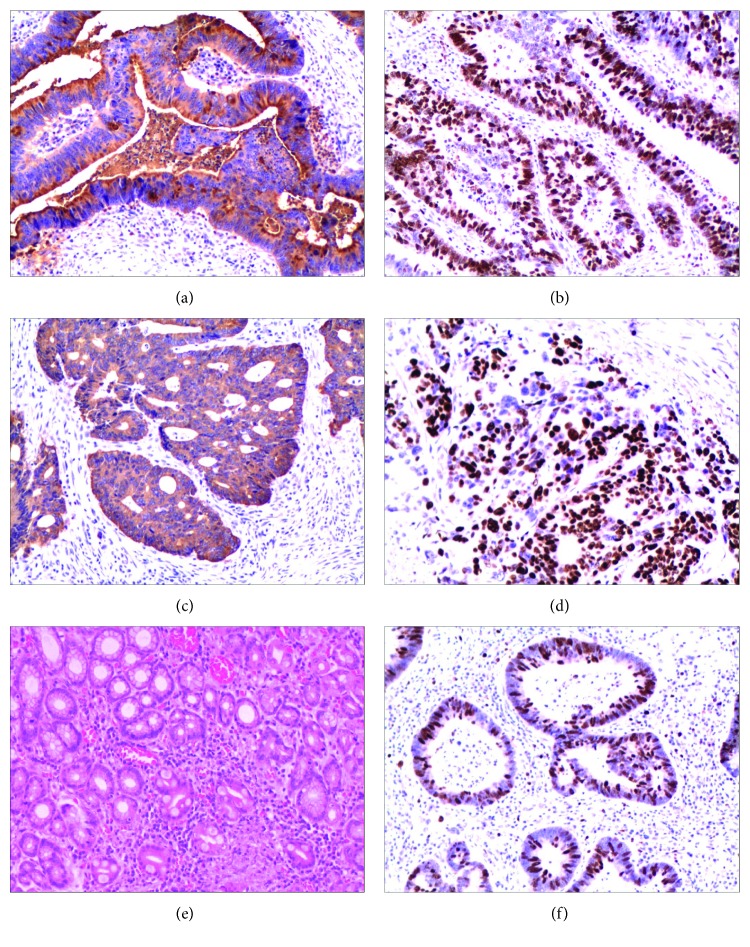
The PIWIL1 was detected by immunohistochemistry. (a and b) PIWIL1 expression in colon cancer tissue. (c and d) PIWIL1 expression in rectum cancer tissue. (e and f) PIWIL1 expression in adjacent tissue. (a, c, and e) Cytoplasm staining of PIWIL1. (b, d, and f) Nucleus staining of PIWIL1.

**Figure 2:**
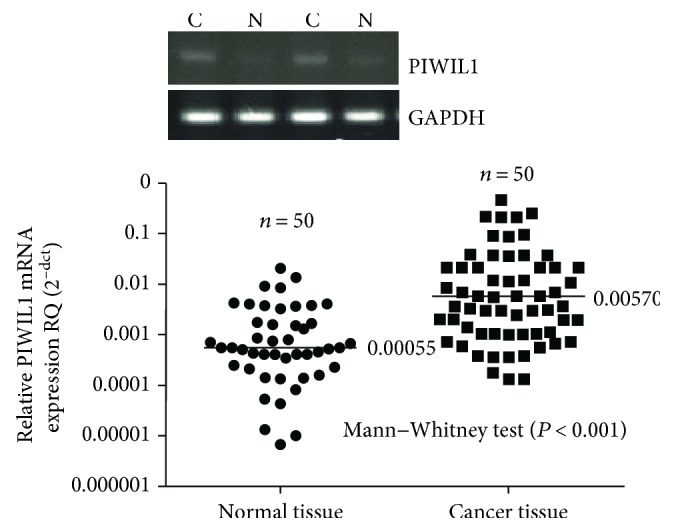
The PIWIL1 mRNA was detected by RT-PCR.

We discovered from the immunohistochemical results that PIWIL1 was mainly located in the cytoplasm of the tumor cells; a small proportion of PIWIL1 was also expressed in the nucleus (Figure 1). In these 110 cases of CRC tissues, 13 samples were found in PIWIL1 protein common expressed in the cytoplasm and nucleus of the tumor cells. That is to say, PIWIL1 protein is only present in cytoplasm of the tumor cells in another 97 samples. The high expression rate of PIWIL1 in the cancer tissue was 54.2% (64/110), which was obviously higher than that in the corresponding adjacent tissue (40.9%, 45/110), with the difference being of statistical significance (*χ*^2^ = 4.053, *P* = 0.044). In order to further verify the abovementioned research results, we randomly selected 50 specimens and quantitatively determined the content of PIWIL1 mRNA in the cancer tissue and adjacent tissue, the results of which suggested that the content of PIWIL1 mRNA in cancer tissue was markedly higher than that in the adjacent tissues (*P* < 0.001, Figure 2).

### 3.2. Association of PIWIL1 Expression with Clinicopathological Features in CRC Patients

The 110 CRC cases were divided into a high PIWIL1 expression and low expression group in accordance with the immunohistochemical evaluation criteria. In the meantime, we analyzed the relationship between the expression of PIWIL1 and the clinicopathological characteristics of CRC patients. The expression of PIWIL1 was closely related to the tumor differentiation degree, infiltration depth, lymphovascular invasion, lymph node metastasis, and TNM stage (*P* < 0.05). However, there was no relationship between PIWIL1 expression levels and age, sex, tumor size, or location (*P* > 0.05). The characteristics are summarized in Table 1.

### 3.3. Survival Analysis of CRC Patients

**Figure 3:**
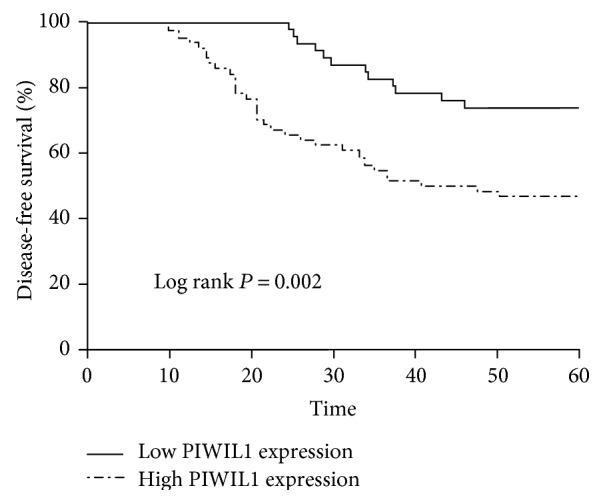
Kaplan-Meier analysis of disease-free survival for colorectal cancer patients according to PIWIL1 expression status.

**Figure 4:**
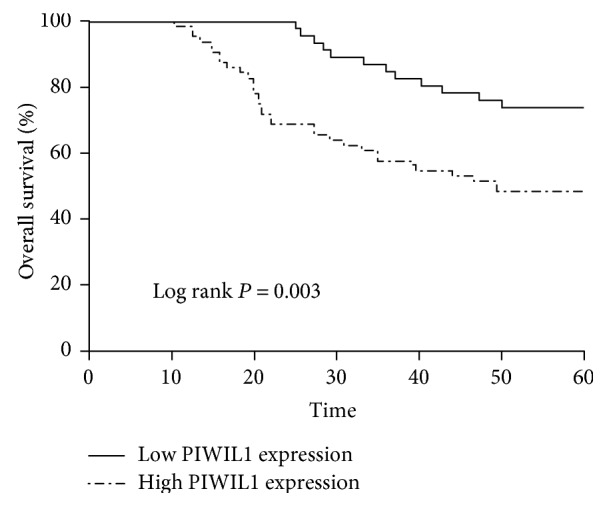
Kaplan-Meier analysis of overall survival for colorectal cancer patients according to PIWIL1 expression status.

In order to investigate the relationship between the expression of PIWIL1 and prognosis of CRC patients, we conducted long-term follow-up of all of the CRC patients and drawn the survival curve. The Kaplan-Meier survival model suggested that the survival time (either DFS or OS) of CRC patients in the high PIWIL1 expression group was notably lower than that in the low PIWIL1 expression group (Table 2 and Figures 3 and 4). A multivariate analysis also showed that PIWIL1 expression was an independent prognostic marker for both DFS and OS of patients with CRC (*P* < 0.05, Table 3).

## 4. Discussion

PIWI is an important member of the human Argonaute protein family. The Argonaute protein is the core component of the RNA-induced silencing complex, which plays an important role in RNA interference, and is closely associated with the genesis of multiple malignancies [9–11]. The Argonaute protein family members can bind with various types of noncoding small RNAs, such as the miRNAs, siRNAs, and PIWI-interacting RNAs [12]. These small RNAs can guide the binding of Argonaute protein with the specific target sequence of mRNA through the complementary base-pairing principle, and thus, it results in the degradation of the target mRNA or inhibits the translation of the target mRNA, producing specific gene silencing effects, participating in a series of biological processes [13, 14]. Its specific PIWI domain has nuclease activity and a functional domain that is highly conservative is located around its C-terminus [15]. PIWI regulates gene expression and takes part in all kinds of biological processes mainly through specific binding with PIWI-interacting RNAs, and its precise regulatory mechanisms include gene silencing, transposon silencing, translation inhibition, and epigenetic changes. PIWIL1 is closely associated with biological behaviors such as proliferation, apoptosis, adhesion, metastasis, and chemotherapy resistance of tumor cells [16].

The chromosomal location of PIWIL1 is 12q24.33 and the protein has 861 amino acid residues, with a molecular weight of 98.6 kDa [17]. PIWIL1 is extensively expressed in human tissues, including prostate, ovary, brain, liver, heart, kidney, and skeletal muscle, and it plays a crucial role in the self-renewal of human stem cells and RNA interference; in addition, it affects the proliferation of cancer cells [17–19]. In this study, we detected the expression of PIWIL1 in CRC tissues, to investigate the relationship between the expression of PIWIL1 and the clinicopathological characteristics of CRC patients.

First, we detected the expression of PIWIL1 in CRC tissue sections, using an immunohistochemical method. We found that PIWIL1 was mainly located in the cytoplasm of the CRC tumor cells. The statistical analysis showed high expression of PIWILI in tumor tissue. Secondly, the quantitative results also revealed that the content of PIWIL1 mRNA in CRC tissue was notably higher than that in the paracarcinoma tissue. We analyzed the relationship between the expression of PIWIL1 and the clinicopathological characteristics of CRC patients. The expression of PIWIL1 was closely related to the tumor differentiation degree, infiltration depth, lymphovascular invasion, lymph node metastasis, and TNM stage. The results suggesting that PIWIL1 might promote the growth, proliferation, and invasion of CRC tumor cells. There are some other studies about PIWIL1 expression status and CRC. Litwin et al. [20] found that higher mRNA levels of PIWIL1 mRNA were measured in CRC tissues compared to those in the corresponding noncancerous samples. There results indicate a reciprocal regulation between PIWIL1 and some cancer stem cells markers in CRC. In the other study, the scholars have observed PIWIL1 mRNA expression was significantly associated to the depth of tumor invasion and the stage of tumorigenesis progression of CRC [21]. Liu et al.'s study showed that high PIWIL1 expression means worse prognosis in CRC patients with negative lymph node [22]. Our data presented in this article supports previously published studies on the impact of PIWIL1 expression in CRC. Although similar to the above findings, our study also found that high PIWIL1 expression levels were closely related to the tumor differentiation degree and lymphovascular invasion of CRC patients. We think that difference of experimental conditions and research objects (such as racial differences), and research bias, may be the cause of the differences in results. However, these were in vivo research results, which need to be further verified through in vitro experiments.

We found through the long-term follow-up of the 110 cases of CRC that the survival time of CRC patients with high PIWIL1 expression was shorter than that of CRC patients with low PIWIL1 expression. The multifactor regression analysis showed that high PIWIL1 expression was one of the important indicators that independently predicted poorer prognosis for CRC patients. We reviewed and analyzed the literature regarding the relationship between PIWIL1 and malignancies on the basis of the results from this study. The possible mechanisms of action of PIWIL1 promoting the tumor progression might be as follows [23–27]: (1) PIWIL1 and the cell proliferation marker Ki-67 had similar expression patterns in the gastric cancer cells, and interference by PIWIL1 RNA could cause cell cycle arrest at the G2/M stage in gastric cancer cells. (2) PIWIL1 silenced CDKI by increasing CDKI methylation, which had an antitumor effect, and eventually resulted in the genesis of osteosarcoma. (3) PIWIL1 was found to be the target gene of the Ras-associated domain family protein 1 C in a lung cancer model. The Ras-associated domain family protein 1 C could induce the phosphorylation of the cancer cell ERKl/2 and thus activate the MEK-ERKl/2 signaling pathway and upregulate the expression of PIWIL1, resulting in the unlimited self-renewal of the tumor stem cells, thereby promoting the genesis and development of a tumor.

## 5. Conclusions

In conclusion, high PIWIL1 expression suggests a poor prognosis for CRC patients and PIWIL1 can serve as an important molecular marker for predicting the prognosis of CRC patients. However, its precise mechanism remains unclear and needs to be illustrated through in-depth research. We believe that blocking PIWIL1-associated signal transduction pathways can delay tumor growth, proliferation, infiltration, and metastasis.

## Figures and Tables

**Table 1:** Association between PIWIL1 overexpression and clinicopathologic characteristics.

Characteristics	Number	PIWIL1 expression		*P* ^a^
		Low (%)	High (%)	
Age (years)				0.477
<60	57	22 (47.8)	35 (54.7)	
≥60	53	24 (52.2)	29 (45.3)	
Sex				0.350
Male	56	21 (45.7)	35 (54.7)	
Female	54	25 (54.3)	29 (45.3)	
Tumor size				0.528
<5 cm	44	20 (43.5)	24 (37.5)	
≥5 cm	66	26 (56.5)	40 (62.5)	
TNM stage				0.007
I	23	14 (30.4)	9 (14.1)	
II	42	19 (41.3)	23 (35.9)	
III	34	11 (23.9)	23 (35.9)	
IV	11	2 (4.3)	9 (14.1)	
Differentiation				0.017
Well	31	18 (39.1)	13 (20.3)	
Moderate	47	19 (41.3)	28 (43.8)	
Poor	32	9 (19.6)	23 (35.9)	
Infiltrate depth				0.002
T1 + T2	43	26 (56.5)	17 (26.6)	
T3 + T4	67	20 (43.5)	47 (73.4)	
Lymphovascular invasion				0.012
No	61	32 (69.6)	29 (45.3)	
Yes	49	14 (30.4)	35 (54.7)	
Lymph node metastasis				0.032
No	71	35 (76.1)	36 (56.3)	
Yes	39	11 (23.9)	28 (43.8)	
Tumor location				0.383
Rectum	58	22 (47.8)	36 (56.3)	
Colon	52	24 (52.2)	28 (43.8)	

LNM: lymph node metastasis; TNM: tumor node metastasis; ^a^chi-square test.

**Table 2:** Univariate survival analysis of OS and DFS in 110 patients with colorectal cancer.

Characteristics	DFS			OS		
	Mean *±* SE (months)	95% CI	*P* ^a^	Mean *±* SE (months)	95% CI	*P* ^a^
Age (years)			0.421			0.332
<60	43.79 ± 2.57	38.75–48.84		44.50 ± 2.50	39.60–49.40	
≥60	48.05 ± 2.20	43.74–52.36		49.50 ± 2.07	45.45–53.55	
Sex			0.931			0.808
Male	45.01 ± 2.52	40.07–49.94		45.91 ± 2.43	41.14–50.68	
Female	46.71 ± 2.32	42.17–51.25		47.94 ± 2.21	43.60–52.27	
Tumor size			0.597			0.495
<5 cm	46.93 ± 2.64	41.76–52.09		47.76 ± 2.56	42.75–52.77	
≥5 cm	45.12 ± 2.25	40.71–49.53		46.34 ± 2.16	42.11–50.56	
TNM stage			<0.001			<0.001
I	55.49 ± 2.66	50.27–60.71		55.77 ± 2.58	50.71–60.82	
II	54.44 ± 1.73	51.06–57.83		54.99 ± 1.62	51.81–58.16	
III	33.52 ± 2.42	28.77–38.27		35.82 ± 2.43	31.06–40.59	
IV	27.43 ± 6.19	15.29–39.56		28.19 ± 6.04	16.36–40.02	
Differentiation			<0.001			<0.001
Well	54.44 ± 2.17	50.19–58.69		55.13 ± 2.00	51.20–59.05	
Moderate	49.47 ± 2.03	45.49–53.46		50.83 ± 1.88	47.14–54.51	
Poor	31.15 ± 3.32	24.65–37.65		32.12 ± 3.22	25.81–38.43	
Infiltrate depth			<0.001			<0.001
T1 + T2	53.80 ± 2.19	49.50–58.10		53.99 ± 2.13	49.81–58.17	
T3 + T4	40.74 ± 2.23	36.37–45.10		42.36 ± 2.16	38.12–46.60	
Lymphovascular invasion			<0.001			<0.001
No	57.04 ± 1.30	54.49–59.60		57.19 ± 1.25	54.73–59.65	
Yes	31.90 ± 2.25	27.50–36.30		34.10 ± 2.29	29.61–38.60	
LNM			<0.001			<0.001
No	52.19 ± 1.74	48.79–55.59		53.02 ± 1.65	49.78–56.26	
Yes	34.28 ± 2.85	28.69–39.87		35.78 ± 2.77	30.35–41.22	
Tumor location			0.090			0.067
Rectum	43.48 ± 2.35	38.87–48.09		44.86 ± 2.25	40.45–49.27	
Colon	48.48 ± 2.46	43.66–53.29		49.19 ± 2.39	44.51–53.87	
PIWIL1 expression			0.002			0.003
Low	52.90 ± 1.83	49.31–56.50		53.53 ± 1.72	50.16–56.90	
High	40.77 ± 2.45	35.97–45.57		42.14 ± 2.38	37.48–46.81	

OS: overall survival; DFS: disease-free survival; SE: standard error; CI: confidence interval; LNM: lymph node metastasis; TNM: tumor node metastasis; ^a^log-rank test.

**Table 3:** Cox regression analysis of OS and DFS in 110 patients with colorectal cancer.

Characteristics	DFS			OS		
	HR	95% CI	*P* ^a^	HR	95% CI	*P* ^a^
TNM stage			0.048			0.031
I						
II	1.316	0.265–6.545	0.737	1.064	0.210–5.382	0.940
III	3.323	0.761–14.514	0.110	3.007	0.702–12.876	0.138
IV	4.510	1.906–12.462	0.046	4.502	1.915–12.146	0.044
Differentiation			<0.001			<0.001
Well						
Moderate	0.808	0.260–2.511	0.713	0.828	0.268–2.558	0.743
Poor	4.151	1.941–9.493	0.002	4.401	2.017–10.315	0.002
Lymphovascular invasion			0.006			0.009
No						
Yes	2.756	1.606–8.082		2.029	1.714–9.750	
PIWIL1 expression			0.002			0.005
Low						
High	3.227	1.563–6.662		2.803	1.374–5.717	

OS: overall survival; DFS: disease-free survival; HR: hazard ratio; CI: confidence interval; TNM: tumor node metastasis; ^a^Cox regression test.
